# Quarter-Wave Plate Metasurfaces for Generating Multi-Channel Vortex Beams

**DOI:** 10.3390/nano14040374

**Published:** 2024-02-17

**Authors:** Ziheng Zhang, Manna Gu, Guosen Cui, Yuxiang Zhou, Teng Ma, Kaixin Zhao, Yunxiao Li, Chunxiang Liu, Chuanfu Cheng, Li Ma

**Affiliations:** 1 School of Physics and Electronics, Shandong Normal University, Jinan 250014, China; zzh1074101434@163.com (Z.Z.); gumanna1996@163.com (M.G.); cuiguosen6688@163.com (G.C.); yx_zhou07@163.com (Y.Z.); mateng_mt1320@163.com (T.M.); 13011611955@163.com (K.Z.); yunxiaoli0926@163.com (Y.L.); liuchunxiang@sdnu.edu.cn (C.L.); 2 Department of Physics, Changzhi University, Changzhi 046011, China

**Keywords:** metasurface, vortex beams, quarter-wave plate, multi-channel

## Abstract

Metasurfaces of quarter-wave plate (QWP) meta-atoms have exhibited high flexibility and versatile functionalities in the manipulation of light fields. However, the generation of multi-channel vortex beams with the QWP meta-atom metasurfaces presents a significant challenge. In this study, we propose dielectric metasurfaces composed of QWP meta-atoms to manipulate multi-channel vortex beams. QWP meta-atoms, systematically arranged in concentric circular rings, are designed to introduce the modulations via the propagation phase and geometric phase, leading to the generation of co- and cross-polarized vortex beams in distinct channels. Theoretical investigations and simulations are employed to analyze the modulation process, confirming the capability of QWP meta-atom metasurfaces for generating the multi-channel vortex beams. This study presents prospective advancements for the compact, integrated, and multifunctional nanophotonic platforms, which have potential applications in classical physics and quantum domains.

## 1. Introduction

Vortex beams represent a specialized category of optical beams carrying orbital angular momentum (OAM). The intensity profile of the vortex beam is characterized by a circular distribution with a dark core, while the phase presents as a rotational distribution with an indefinite phase at the center [1]. Vortex beams have found extensive applications across various scientific fields, including but not limited to particle manipulation [2], optical communication [3], quantum entanglement [4], and data storage [5]. The generation of the vortex beams plays a crucial role in advanced developments, and presently, the common methods for generating vortex beams involve the spatial light modulators [6], spiral phase plate [7], and *q* plates [8]. However, these methods inevitably need bulky optical elements, intricate operational procedures, and strict optical modulations. This stands in contrast to the prevailing trend of device miniaturization, thereby increasing challenges for their integrations in compact and miniaturized optical systems.

Metasurfaces are inhomogeneous and anisotropic surface structures consisting of arrays of subwavelength dielectric or metallic meta-atoms [9]. Based on the interactions of light with these structures, metasurfaces enable precise control over the amplitude, phase, and polarization of the output light [10,11,12]. Furthermore, due to the compact dimensions and lightweight nature of metasurfaces, they offer a profitable approach for the development of integrated and multifunctional optical systems. Metasurfaces have demonstrated considerable potential applications across diverse fields, including three-dimensional imaging [13,14], vector beams (VBs) [15,16,17], metalenses [18], and optical holography [19]. Significantly, metasurfaces have become a highly effective tools for the generation of vortex beams at the nanoscale [20,21,22,23]. As exemplified by the study of Liu et al. [22], metasurfaces have been employed as quantum emitters to generate single-photon linearly polarized vortex beams. In a relevant study, Liu et al. [23] utilized dielectric metasurfaces to produce broadband perfect vortex beams. In the prevalent studies, the predominant designs of metasurfaces employed half-wave plate (HWP) meta-atoms to induce chiral modulation in incident light. However, the metasurfaces of HWP meta-atoms exclusively achieve the generation of cross-polarized components in the output field. In contrast, quarter-wave plate (QWP) meta-atoms utilize both propagation and geometric phases concurrently to control the co- and cross-polarized components [24]. This characteristic enables the metasurfaces to be more flexible in wavefield manipulations, facilitating multifunctional applications in diverse areas [25]. Therefore, in recent years, the investigations of QWP meta-atom metasurfaces have aroused great interest. Examples include these metasurfaces to generate focused VBs [26] and linear-to-circular polarization conversion [10,27,28], realize multifocal vectorial holography, and enable wavelength-multiplexed dual-image display and encryption [29]. Consequently, the exploration of multi-channel vortex light fields generated by utilizing QWP meta-atom metasurfaces represents a highly significant research area.

In this study, we have designed metasurfaces with QWP meta-atoms to generate multi-channel vortex beams. The metasurfaces are constructed by arranging QWP meta-atoms in concentric circular rings. The phase profiles of the wavefield modulated by meta-atoms comprise both a propagation phase *φ*_1_ and a geometric phase *φ*_2_. The propagation phase *φ*_1_ is designed to contain both a hyperbolic phase and a propagation vortex phase, whereas the geometric phase *φ*_2_ is structured to contain a gradient phase and a geometric vortex phase. Systematic variations in the dimensions and the orientation angles of meta-atoms enable the precise manipulation of phases *φ*_1_ and *φ*_2_ in the output field. In the case of circularly polarized incident light, the chirality-dependence of the geometric phase acting on the cross-polarization component yields vortex beams with different orders and different deflecting directions in the cross-polarized channel. The co-polarized component is only subject to the propagation phase, leading to the generation of a non-deflected vortex beam in the co-polarized channel. When linearly polarized light illuminates the metasurface, it generates two vortex beams with distinct orders in the two cross-polarized channels and a linearly polarized vortex beam in the co-polarized channel. Our work theoretically analyzed the multi-channel vortex fields generated by QWP meta-atom metasurfaces. Subsequently, by employing the finite-difference time-domain (FDTD) method, we conducted simulations to characterize the optical properties of the output light field. This study provides a new perspective for manipulating vortex fields through QWP meta-atom metasurfaces. The results make substantial contributions to the progress of compact, integrated, and multifunctional nanophotonic platforms. These advancements hold significant implications for applications related to vortex phenomena, particularly in the fields of particle trapping and information communication.

## 2. Principles of the Metasurface Design

### 2.1. Overview of Principle

**Figure 1:**
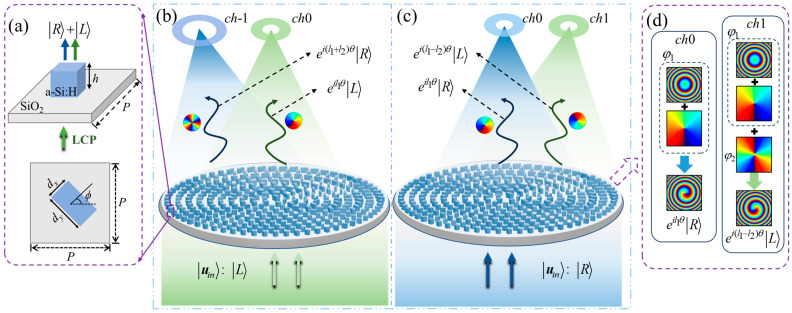
The schematic generations of multi-channel vortex beams based on dielectric QWP meta-atom metasurfaces. (**a**) A meta-atom and the transmitted wavefield under the LCP illumination, with corresponding parameters: *ϕ* is the orientation angle of meta-atom, *d_x_* is the length, *d_y_* is the width, *h* is the height fixed at 480 nm, and *P* is the lattice constant set to 380 nm. (**b**,**c**) Generations of multi-channel vortex beams by metasurfaces with distinct vortex states in distinct channels under RCP and LCP illuminations. (**d**) Schematics of the phase profiles of the propagation phase, geometric phase, and output phase profiles in the QWP metasurface design.

Figure 1 schematically illustrates the generation of the multi-channel vortex fields by QWP meta-atom metasurfaces. The side and top views of a meta-atom are presented in Figure 1a, featuring a rectangular dielectric QWP-nanopillar of a-Si:H. The height *h* of the meta-atom remains constant, while the parameters of length *d_x_*, width *d_y_*, and orientation angle *ϕ* are variable. Meta-atoms are systematically arranged on the substrate of fused silica SiO_2_ with a lattice constant of *P*, constituting the metasurface. Figure 1b,c show the complete depictions of the constructed metasurfaces, as well as schematic representations for generating multi-channel vortex beams under left circularly polarization (LCP) and right circular polarization (RCP) illuminations. As exemplified in Figure 1b, when the LCP, denoted as *u_in_* = |*L* > = [1 *i*]*^T^*, is the illuminating light, the metasurface modulates and constitutes the output field with both the co-polarized component |*L* > = [1 *i*]*^T^* and cross-polarized component |*R* > = [1 − *i*]*^T^*, where *T* denotes the matrix transpose. In Figure 1d, the phase profiles of propagation phase *φ*_1_ and geometric phase *φ*_2_ within the QWP meta-atom metasurface are illustrated. These profiles exemplify the behavior of the metasurface under RCP illumination, as demonstrated in Figure 1c. In both channels *ch*0 and *ch*1, the propagation phases *φ*_1_ encompasses the hyperbolic phase and propagation vortex phase, as depicted in the top two phase maps in each channel illustration. Meanwhile, the geometric phase *φ*_2_, carrying the geometric vortex phase and gradient phase, operates only on channel *ch*1 to concurrently modulate the light field alongside the propagation phase. (We note that, to observe clearly the phase profile of the hyperbolic and vortex phases, the gradient geometric phase profile is not shown.) The bottom phase maps present, respectively, the propagation phase profile for *ch*0 and the profile combining the propagation and geometric phases for *ch*1. Based on the modulation requirements of QWP meta-atom metasurfaces, the specifically designed geometric structures ensure that the propagation phase *φ*_1_ constructs the hyperbolic phase profile *φ_f_* to focus the vortex field with a focal length of *f* in channel *ch*0. Meanwhile, the geometric phase *φ*_2_ configurates the gradient phase *φ_d_* to deflect the light field towards channels *ch*1 and *ch*-1.

Because the co-polarized component is only imparted with propagation phase *φ*_1_, its wavefront is non-deflected and focuses at the focal plane. This phenomenon is observable through the vortex states of $e^{il_{1}\theta }{|L\rangle }_{ch0}$ and $e^{il_{1}\theta }{|R\rangle }_{ch0}$ in channel *ch*0 in Figure 1b,c, where *l*_1_ is the order of vortex beams and *θ* denotes the azimuthal angle coordinate of the meta-atom. The cross-polarized component generated by the metasurface concurrently receives the modulations from both propagation phase *φ*_1_ and geometric phase *φ*_2_. Consequently, the topological charge of the cross-polarized vortex beam is the superposition of the orders contributed by each of these phases. In Figure 1b,c, the vortex states of $e^{i(l_{1}+l_{2})\theta }{|R\rangle }_{ch-1}$ and $e^{i(l_{1}-l_{2})\theta }{|L\rangle }_{ch1}$ in channels *ch*-1 and *ch*1 are generated through the utilization of cross-polarized components.

As shown in Figure 1b, under LCP illumination, the co-polarized component produces a vortex state of $e^{il_{1}\theta }{|L\rangle }_{ch0}$ in the center of channel *ch*0, while the cross-polarized component presents a RCP vortex state of $e^{i(l_{1}+l_{2})\theta }{|R\rangle }_{ch-1}$ with a deflection angle to the left, directed towards channel *ch*-1. In the case of RCP illumination, the chirality-dependence of the geometric phase results in a reversal of the sign of *φ*_2_. As depicted in Figure 1c, the cross-polarized component generates a LCP vortex state of $e^{i(l_{1}-l_{2})\theta }{|L\rangle }_{ch1}$ with a deflection angle to the right, towards channel *ch*1, while the vortex state of the co-polarized component, denoted as $e^{il_{1}\theta }{|R\rangle }_{ch0}$, stays in the center of channel *ch*0. 

Based on the above analyses, we can logically deduce the phenomenon of linearly polarized light illumination. The linearly polarized light can be reasonably considered as the composition of LCP and RCP components. With linear polarization illuminating the metasurface, the triple-channel vortex beams can be generated, including the circularly polarized vortex beams in channels *ch*-1 and *ch*1, and the linearly polarized vortex beam in channel *ch*0.

On the whole, the polarization of the vortex beam in channel *ch*0 remains consistent with the polarized state of the incident light, while the topological charge of *l*_1_ is exclusively determined by the propagation phase. The states of the vortex beams in channels *ch*-1 and *ch*1 are associated with the polarizations of incident light. Specifically, LCP illumination corresponds to the vortex order of *l*_1_ + *l*_2_ and the leftward deflection angle, whereas RCP illumination corresponds to the vortex order of *l*_1_ − *l*_2_ and the rightward deflection angle.

### 2.2. The Transmitted Light Field of the Meta-Atoms

We consider a rectangular nanopillar meta-atom, characterized by an anisotropic structure with two perpendicular axes of mirror symmetry in length and width. When an incident light linearly polarized along either axis propagates through the meta-atom, the transmitted light field modes polarized along the same axis are produced [30]. The independent phase delays are imparted on these modes, resulting in birefringence, with the two axes serving as the fast and slow axes. Therefore, for a rectangular meta-atom with arbitrary orientation angle *ϕ*, the output field is described by the Jones matrix:

(1) $$J(x,y)=R(-\phi )\left[\begin{array}{cc} e^{i\varphi_{x}} & 0\\ 0 & e^{i\varphi_{y}} \end{array}\right]R(\phi )$$

where $R(\phi )=\left[\begin{array}{cc} \cos\phi & -\sin\phi \\ \sin\phi & \cos\phi \end{array}\right]$ is the rotation matrix, and *φ_x_* and *φ_y_* are the phase delays of the wavefield modes polarizing along the symmetry axes, respectively. In this context, we have made the assumption that the transmittances for the two modes in linear polarizations are equal and approximately unity.

Based on the Jones matrix provided above and the fundamental principles governing the interaction between circularly polarized light and a QWP, we can proceed to calculate and obtain the relevant phase and angle data of meta-atoms. When the QWP meta-atom is illuminated with LCP and RCP lights, the phases of the cross-polarized light fields are given as *φ_L_*(*x*, *y*) and *φ_R_*(*x*, *y*); then, the propagation phase and orientation angle of the meta-atom are derived as follows:

(2) $$\varphi_{x}(x,y)=\varphi_{L}(x,y)+\varphi_{R}(x,y)/2+\pi /2$$

(3) $$\varphi_{y}(x,y)=\varphi_{L}(x,y)+\varphi_{R}(x,y)/2+\pi$$

(4) $$\phi (x,y)=[\varphi_{L}(x,y)-\varphi_{R}(x,y)]/4$$

Under the illumination of |*L* > = [1 *i*]*^T^*, the output field becomes linearly polarized and it is written as follows:

(5) $$u_{out}=e^{i(\varphi_{x}+\phi )}\left(\begin{array}{c} \cos(\phi -\frac{\pi }{4})\\ \sin(\phi -\frac{\pi }{4}) \end{array}\right)$$

We adopt the simple designs of the metasurfaces by arranging phase profiles based on the propagation phase and geometric phase of meta-atoms to realize different functionalities. Specifically, the propagation phase *φ*_1_ = *φ_x_* contains the phase profiles *φ_f_* and *φ_h_*_1_, where *φ_f_* represents a hyperbolic phase profile with focal length *f*, and *φ_h_*_1_ is the vortex phase profile. Hence, the expression for *φ*_1_ can be written as follows:

(6) $$\varphi_{1}=2\pi (f-\sqrt{r^{2}+f^{2}})/\lambda +l_{1}\theta$$

where $2\pi (f-\sqrt{r^{2}+f^{2}})/\lambda =\varphi_{f}$ is the hyperbolic phase for focusing, $r=\sqrt{x^{2}+y^{2}}$ is the radius, *f* is the focal length denoted as the distance between the metasurface and the observation plane, and *l*_1_*θ* = *φ_h_*_1_ is the vortex phase with topological charge *l*_1_.

The geometric phase *φ*_2_ contains the phase profiles *φ_d_* and *φ_h_*_2_, where *φ_d_* represents the gradient phase profile with a deflection angle *α*, and *φ_h_*_2_ is the vortex phase profile with topological charge *l*_2_. Additionally, the geometric phase *φ_h_*_2_ depends on the orientation angle *ϕ* of the meta-atom. *ϕ* can be written as follows:

(7) $$\phi =\phi_{0}+m\theta +\pi x\sin\alpha /\lambda$$

where *ϕ*_0_ denotes the initial orientation angle of the meta-atoms at *θ* = 0, and *m* is the rotational order of the meta-atoms. The designed meta-atoms have the established relationships, specifically, *l*_2_ = 2 *m* and *φ*_2_ = 2*ϕ*. The initial orientation angles of the meta-atoms are set as *ϕ*_0_ = 0, to ensure that *φ_h_*_2_ = *l*_2_*θ* = 2 *mθ* corresponds to the vortex phase and *φ_d_* = 2*πxsinα*/*λ* corresponds to the gradient geometric phase. This design induces a deflection angle *α* to the cross-polarized component, with the direction of the deflected light related to the chirality of the incident circular polarization.

Thus, when the illumination is circularly polarized light |*L* > = [1 *i*]*^T^*, we can express the output field of the meta-atom as follows:

(8) $$\begin{array}{c} u_{out}(r,\theta )=u_{ch0}+u_{ch-1}\\ \;=\frac{1}{2}e^{i\varphi_{1}}\left[\begin{array}{c} 1\\ i \end{array}\right]+\frac{1}{2}e^{i\varphi_{1}}e^{i\varphi_{2}}\left[\begin{array}{c} 1\\ -i \end{array}\right] \end{array}$$

By substituting Equations (6) and (7) into Equation (8), we can obtain the output field:

(9) $$u_{out}(r,\theta )=\frac{1}{2}e^{i\varphi_{f}}(e^{il_{1}\theta }|L\rangle +e^{i(l_{1}+l_{2})\theta }e^{i\varphi_{d}}|R\rangle )$$

Equation (9) demonstrates that the co-polarized component generates a vortex with topological charge *l*_1_ in channel *ch*0. At the same time, the cross-polarized component, influenced by both the geometric vortex phase and the propagation vortex phase, produces a vortex beam carrying order *l_ch_*_-1_ = *l*_1_ + *l*_2_, with a deflection angle *α* in channel *ch*-1.

Similarly, under the |*R*> = [1 − *i*]*^T^* illumination, the transmitted light field of the meta-atom is written as follows:

(10) $$u_{out}=e^{i(\varphi_{x}-\phi )}\left(\begin{array}{c} \cos(\phi +\frac{\pi }{4})\\ \sin(\phi +\frac{\pi }{4}) \end{array}\right)$$

Subsequently, the expression of the light field emitted by the meta-atom is obtained as follows:

(11) $$\begin{array}{l} u_{out}(r,\theta )=u_{ch0}+u_{ch1}\\ \;\frac{1}{2}e^{i\varphi_{1}}\left[\begin{array}{c} 1\\ -i \end{array}\right]+\frac{1}{2}e^{i\varphi_{1}}e^{-i\varphi_{2}}\left[\begin{array}{c} 1\\ i \end{array}\right] \end{array}$$

Based on Equations (6), (7), and (11), the output field of the meta-atom under RCP illumination can be expressed as follows:

(12) $$u_{out}(r,\theta )=\frac{1}{2}e^{i\varphi_{f}}(e^{il_{1}\theta }|R\rangle +e^{i(l_{1}-l_{2})\theta }e^{-i\varphi_{d}}|L\rangle )$$

Equation (12) indicates that the co-polarized component continues to yield a vortex with topological charge *l*_1_ in channel *ch*0. Because of the chirality-dependence of geometric phase, both the geometric gradient phase and the geometric vortex phase take a sign reversal. Consequently, when the meta-atom is illuminated by |*R* > = [1 − *i*]*^T^*, its cross-polarized component produces a vortex beam with an order *l_ch_*_1_ = *l*_1_ − *l*_2_ and a deflection angle of −*α* in channel *ch*1.

When the incident light is linear polarization, as the linearly polarized light contains the LCP and RCP components, the output light field will concurrently present the vortex beams with orders *l_ch_*_1_ and *l_ch_*_-1_ in channels *ch*-1 and *ch*1, respectively. Simultaneously, a linearly polarized vortex beam is generated in channel *ch*0, maintaining the same polarization as the incident light.

## 3. Simulation Results

The FDTD method stands out as a specialized tool tailored for photonics and nanophotonics, offering a comprehensive suite of professional simulation capabilities in photonics. In contrast to several conventional numerical approaches like COMSOL and Computer Simulation Technology (CST), FDTD simulations excel in precision, flexibility, and convenience, particularly in conducting research on metasurfaces. Therefore, we chose the FDTD method to calculate the transmitted light field of the QWP meta-atom metasurfaces. Based on the above principles, we employed the FDTD method to conduct the simulations of multi-channel vortex beams generated by the designed QWP meta-atom metasurfaces. In practice, we employed the rectangular nanopillars composed of dielectric material a-Si:H as the meta-atoms, with a fixed height of *h* = 480 nm. The lattice constant *P*, representing the radial and azimuthal spacing between adjacent meta-atoms, was set to 380 nm. At the wavelength of *λ* = 800 nm, the refractive index and extinction coefficient were *n* = 3.744 and *κ* = 0.000, respectively. The substrate was composed of fused silica SiO_2_. When the meta-atom was illuminated with *x*- and *y*-linearly polarized light, we performed two-dimensional parametric sweeps over the output field and obtained the phases and amplitudes versus the length *d_x_* and width *d_y_* of the meta-atoms. As listed in Table 1, eight QWP meta-atoms were selected. The propagation phases *φ_x_* and *φ_y_* are given in the second to third rows, and the transmission amplitudes *T_xx_* and *T_yy_* are reported in the fourth to fifth rows. In Table 1, we may see that for some meta-atoms (such as 1, 2, 5, 6, and 7), *φ_y_* is greater than *φ_x_*, while for some meta-atoms (such as 3, 4, and 8), *φ_y_* is smaller than *φ_x_*. This originates from the utilization of the QWP condition |*φ_x_* − *φ_y_* | = *π*/2 for selecting the meta-atoms, which includes the two cases of *φ_x_* − *φ_y_* = *π*/2 and *φ_x_* − *φ_y_* = −*π*/2. In selecting the required QWP meta-atoms from the results of the parameter sweep using the FDTD method, *φ_x_* is taken as the propagation phase. For the meta-atoms to be selected, the values of *φ_x_* should cover the phase range of 0 − 2*π*, and *φ_x_* should be linearly increased across the eight meta-atoms. To ensure this linear increase, which provides convenience for configurating the phase profiles in our metasurface design, the meta-atoms are numbered in order according to the values of *φ_x_* in Table 1. While based on phase retardation |*φ_x_* − *φ_y_*| = *π*/2, the selected *φ_y_* maintaining a difference with *φ_x_* of ±*π*/2 will satisfy the QWP condition. *T_xx_* and *T_yy_* remain close to 1, supporting a good light transmission. 

In metasurface design, we arranged meta-atoms based on the principles outlined in Equation (6) for the propagation phase and Equation (7) for the orientation angle. Initially, we established the focal length f and the topological charge of the propagation vortex from Equation (6) to define the propagation phase profile of the metasurface. Subsequently, we determined the phase requirements for each point on the metasurface and selected the optimal meta-atom size from the eight available options to satisfy these conditions. Similarly, we used Equation (7) to set the beam deflection angle and the geometric topological charge, thereby determining the orientation angle *ϕ* for each meta-atom. Consequently, we combined the required size and orientation angle *ϕ* of each meta-atom at every point to construct the entire metasurface.

In the process of FDTD simulation, we initially conducted two-dimensional parametric sweeps of the meta-atoms, with a cell size of 0.38 μm × 0.38 μm × 4 μm. The incident light was set as a linearly polarized light to separately illuminate the meta-atoms along the *x*- and *y*-directions, with corresponding boundary conditions of “Anti-symmetric”, “Symmetric”, and “Perfectly Matched Layer (PML)”, respectively. Subsequently, all parameters, encompassing the meta-atom transmission phase, geometric phase, meta-atom size, and metasurface dimensions, were obtained. Based on these parameters, we could generate the metasurface within the FDTD simulation framework. Finally, comprehensive FDTD simulations of the entire metasurface were performed, employing a computational range of 80 μm × 80 μm × 3 μm and boundary conditions of PML. The two metasurface samples, denoted as samples 1 and 2, possessed an identical focal length *f* = 95 μm and had a common diameter of 76 μm. Unless otherwise noted, the parameters of the metasurface remained consistent with those specified in the previous Section. In the simulations, the monitor in FDTD was set at a distance of 1 μm from the metasurface, and the data of the light field were projected to the far-field to achieve the wavefield in the observation plane. 

**Figure 2:**
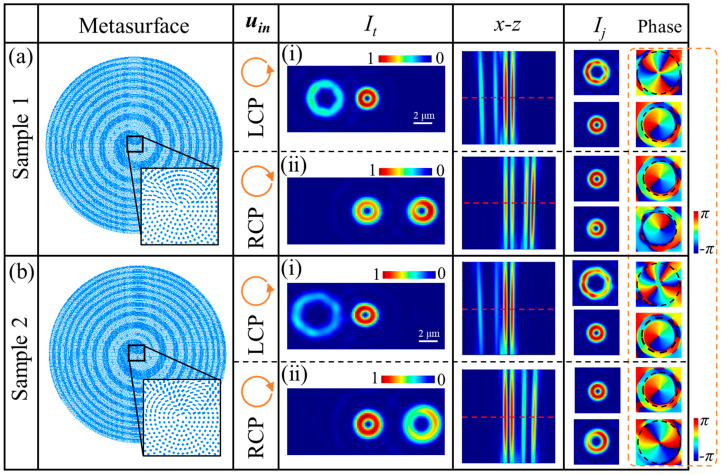
Simulations of the vortex beams based on (**a**) sample 1, and (**b**) sample 2 under panel (i) LCP, and panel (ii) RCP illuminations, respectively. The complete and enlarged images of samples 1 and 2 are displayed in the second column. The patterns in the fourth to sixth columns present the simulated results, including the intensity images of output light fields, the decomposed intensity profiles in the *x*–*z* plane, and the enlarged intensity and corresponding phase distributions, respectively.

Figure 2 shows the simulation results for metasurface samples 1 and 2 under LCP and RCP illuminations. The complete and locally enlarged images of the metasurfaces for the two samples are presented in the second column of Figure 2. The top title row and the corresponding columns display the information of vortex fields, including the incident light polarization state *u_in_*, the total intensity images *I_t_* of vortex beams, the decomposed intensity profiles in the *x–z* plane (with range in the *z*-direction between 90–100 μm), the enlarged and normalized intensity images of three channels *I_j_* (*j* = *ch*-1, *ch*0, or *ch*1), and the phase distributions of vortex beams.

Panels (i) and (ii) in Figure 2a demonstrate total intensity images of the vortex field generated by sample 1 under LCP and RCP illuminations, respectively. *I_j_* are the normalized intensity images locally enlarged around the channel centers. From panel (i) in Figure 2a, it can be observed that the vortex beams with an order *l_ch_*_-1_ = 3 in channel *ch*-1 and an order *l_ch_*_0_ = 1 in channel *ch*0 were generated by sample 1 under LCP illumination. Panel (ii) in Figure 2a shows the generated vortex beams with an order *l_ch_*_1_ = −1 in channel *ch*1 and an order *l_ch_*_0_ = 1 in channel *ch*0 when the sample 1 is illuminated with RCP.

Similarly, panel (i) in Figure 2b illustrates the vortex beams with an order *l_ch_*_-1_ = 4 in channel *ch*-1 and an order *l_ch_*_0_ = 1 in channel *ch*0 when sample 2 is under LCP illumination. In addition, panel (ii) in Figure 2b depicts the vortex beams with sn order *l_ch_*_1_ = −2 in channel *ch*1 and an order *l_ch_*_0_ = 1 in channel *ch*0 with RCP illuminating sample 2.

**Figure 3:**
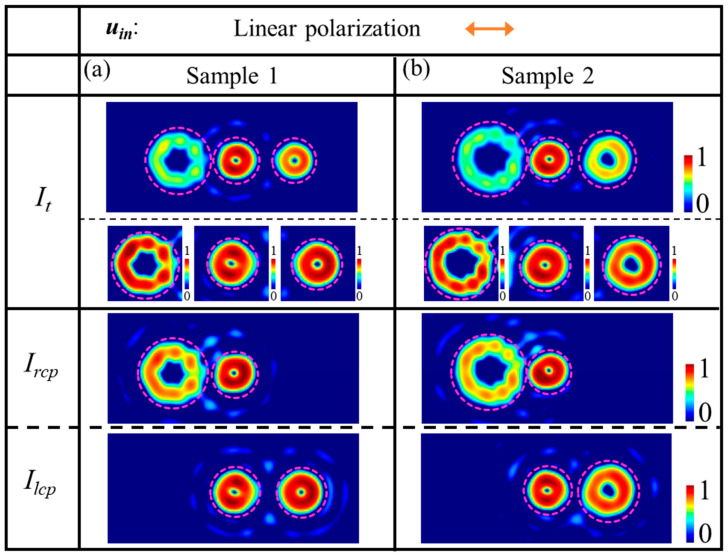
Simulations of the vortex beams based on metasurface samples (**a**) 1, and (**b**) 2, respectively, under linear incident light. The patterns in the third to fifth rows present the simulated results, including the total field intensity images *I_t_*, the RCP component images *I_rcp_*, and the LCP component images *I_lcp_*. In the *I_t_* panel, the first row illustrates the complete intensity field, and the second row shows the normalized and enlarged intensity images with respect to each channel.

Figure 3 shows the simulation results for the metasurface samples 1 and 2 under linearly polarized illumination. The top title row shows the linear polarization state *u_in_* of incident light. The first column indicates the components of the light field, including the total field intensity images *I_t_*, the RCP component images *I_rcp_*, and the LCP component images *I_lcp_*. In the *I_t_* panel, the first row illustrates the complete intensity field, while the second row shows the normalized and enlarged intensity images in distinct channels. Additionally, purple dashed lines are employed to encircle the vortices, to facilitate the identification of corresponding vortices and to assist in locating the images in each channel.

The results presented in Figure 3 illustrate that when sample 1 is illuminated with linearly polarized light, the total field image contains three distinct channels of vortex beams characterized by different orders. Specifically, these channels include the vortex beams with an order *l_ch_*_-1_ = 3 in channel *ch*-1, an order *l_ch_*_0_ = 1 in channel *ch*0, and an order *l_ch_*_1_ = −1 in channel *ch*1. On the other hand, from the components *I_rcp_* and *I_lcp_*, it can be seen that the *ch*0 channel is the superposition of a left-handed circular vortex beam with an order *l_ch_*_0_ = 1 and a right-handed circular vortex beam with an order *l_ch_*_0_ = 1, resulting in a linearly polarized vortex. Similarly, Figure 3b shows different vortex beams generated by sample 2 under linearly polarized illumination. The difference is that the vortex beams in channels *ch*-1 and *ch*1 are *l_ch_*_-1_ = 4 and *l_ch_*_1_ = −2, respectively. In channel *ch*0, it still is a linearly polarized vortex.

The vortex beam images presented in Figure 2 and Figure 3 for samples 1 and 2 reveal a good agreement between simulation results and theoretical derivations. Overall, the quality of vortex beams generated by both samples is deemed satisfactory based on the observed consistency between theoretical predictions and simulated outcomes.

## 4. Discussion and Conclusions

The QWP meta-atom metasurfaces demonstrate the capability to simultaneously manipulate both co-polarized and cross-polarized components within the light field. Through the specific design of the QWP meta-atom metasurfaces, the propagation phase profiles enable the generations of co-polarized vortex beams or the precise focusing of the co-polarized components [27]. Moreover, by configuring the propagation and geometric phase profiles, these metasurfaces can effectively manipulate cross-polarized components to produce multiple functional beams. The designed QWP meta-atom metasurfaces greatly enhance the capabilities of manipulating the phase and polarization.

Compared to existing HWP metasurfaces, the QWP meta-atom metasurface designs provide significantly higher information capacity, particularly in applications like information transmission, thus accelerating advancements in this field. While existing QWP meta-atom metasurfaces typically generate one- [27] or two- [10] channel beams (co-polarized and cross-polarized), our research extends this capability to include the generation of three-channel vortex beams, representing a novel contribution with significant implications.

In this study, we designed the QWP meta-atom metasurfaces to generate multi-channel vortex beams. The metasurfaces were constructed using QWP meta-atoms systematically arranged in concentric circular rings. Based on the designed propagation phase and geometric phase within the QWP meta-atoms, the metasurfaces exhibited the ability to independently modulate the co- and cross-polarized components, leading to the generation of a non-deflected co-polarized vortex and a deflected cross-polarized polarized vortex. The direction of deflection was associated with the polarization states of the incident light, owing to the inherent charity of the geometric phase. Based on the theoretical analyses and FDTD simulations, we demonstrated the feasibility of manipulating the vortex fields via the proposed QWP meta-atom metasurfaces for generating multi-channel beams. This study offers waveplate metasurfaces which can be applied in fields such as high-capacity communications and multi-particle manipulations. The findings hold substantial significance for the miniaturization of optical devices and the integration of optical systems.

## Figures and Tables

**Table 1:** The parameters of meta-atoms.

unit/*N*	1	2	3	4	5	6	7	8
	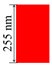	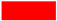	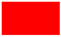	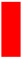	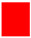	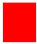	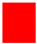	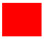
*φ_x_* (*π*)	−0.00669	0.23417	0.47558	0.73053	1.02955	1.22179	1.48022	1.75448
*φ_y_* (*π*)	0.47558	0.73053	−0.00669	0.23417	1.53309	1.75448	1.99000	1.22179
*T_xx_*	0.98469	0.92895	0.94721	0.97474	0.96961	0.98932	1.00000	0.94512
*T_yy_*	0.94721	0.97474	0.98469	0.92895	0.92895	0.94512	0.94373	0.98327

The parameters of propagation phase *φ_x/y_* and transmission amplitudes *T_xx/yy_* corresponding to the eight QWP meta-atoms. *N* denotes the sequential number of the meta-atoms.

## Data Availability

The data that support the findings of this study are available from the corresponding author upon reasonable request.
